# An IoT Measurement System Based on LoRaWAN for Additive Manufacturing

**DOI:** 10.3390/s22155466

**Published:** 2022-07-22

**Authors:** Tommaso Fedullo, Alberto Morato, Giovanni Peserico, Luca Trevisan, Federico Tramarin, Stefano Vitturi, Luigi Rovati

**Affiliations:** 1 Department of Engineering “Enzo Ferrari”, University of Modena and Reggio Emilia, Via P. Vivarelli 10, 41125 Modena, Italy; tommaso.fedullo@unimore.it (T.F.); luigi.rovati@unimore.it (L.R.); 2 Department of Management and Engineering, University of Padova, S. S. Nicola 3, 36100 Vicenza, Italy; 3 National Research Council of Italy, CNR–IEIIT, Via Gradenigo 6/B, 35131 Padova, Italy; alberto.morato@ieiit.cnr.it (A.M.); stefano.vitturi@ieiit.cnr.it (S.V.); 4 Department of Information Engineering, University of Padova, Via Gradenigo 6/B, 35100 Padova, Italy; giovanni.peserico@phd.unipd.it; 5 Autec s.r.l., Via dei Pomari 65, 36030 Caldogno, Italy; 6 Consorzio RFX, Corso Stati Uniti 4, 35127 Padova, Italy; luca.trevisan@igi.cnr.it

**Keywords:** smart sensors, Industrial IoT, LoRa, IoT measurement systems, smart monitoring, battery lifetime

## Abstract

The Industrial Internet of Things (IIoT) paradigm represents a significant leap forward for sensor networks, potentially enabling wide-area and innovative measurement systems. In this scenario, smart sensors might be equipped with novel low-power and long range communication technologies to realize a so-called low-power wide-area network (LPWAN). One of the most popular representative cases is the LoRaWAN (Long Range WAN) network, where nodes are based on the widespread LoRa physical layer, generally optimized to minimize energy consumption, while guaranteeing long-range coverage and low-cost deployment. Additive manufacturing is a further pillar of the IIoT paradigm, and advanced measurement capabilities may be required to monitor significant parameters during the production of artifacts, as well as to evaluate environmental indicators in the deployment site. To this end, this study addresses some specific LoRa-based smart sensors embedded within artifacts during the early stage of the production phase, as well as their behavior once they have been deployed in the final location. An experimental evaluation was carried out considering two different LoRa end-nodes, namely, the Microchip RN2483 LoRa Mote and the Tinovi PM-IO-5-SM LoRaWAN IO Module. The final goal of this research was to assess the effectiveness of the LoRa-based sensor network design, both in terms of suitability for the aforementioned application and, specifically, in terms of energy consumption and long-range operation capabilities. Energy optimization, battery life prediction, and connectivity range evaluation are key aspects in this application context, since, once the sensors are embedded into artifacts, they will no longer be accessible.

## 1. Introduction

In recent decades, the Internet of Things (IoT) has gained ever more visibility, due to its features that allow the interconnection of several heterogeneous “things”, that can be accessed from anywhere, using any kind of connected device [1]. IoT devices are nowadays employed in several fields, such as agriculture [2], environmental monitoring and smart cities [3], smart grids [4], etc. In the industrial context, the adoption of IoT, together with the features of the Industry 4.0 paradigm, is usually referred to as the Industrial Internet of Things (IIoT) [5].

This has opened the way to novel developments in the field of sensor networks. In general, these are required to provide support for distributed measurement functionalities, potentially with the provision of high accuracy levels, continuous measurement capabilities and causality preservation of sensor data, to guarantee enhanced control capabilities, high quality and flexible production. Moreover, in the industrial scenario, sensor data often needs to be conveyed with high reliability and strict timing requirements, such as determinism, bounded latency, and time synchronization. For this purpose, IIoT systems typically adopt specific network technologies, such as fieldbuses, real-time (or industrial) ethernet and industrial Wi-Fi systems [6,7,8].

Nonetheless, for some novel IIoT applications, the rate at which the data is acquired might not be of primary importance, whereas long transmission ranges and low battery consumption may be essential. For these reasons, in the last few years, sensor networks meeting such requirements have been studied and applied. In this context, low-power wide-area networks (LPWANs) have proven to be very effective, because of their beneficial features. The interested reader can refer to [9], where different widespread LPWAN technologies are addressed and compared, with a focus on both their characteristics and on the multitude of scenarios in which they can be applied. Among the available LPWAN protocols for this kind of application, long-range (LoRa) protocols have attracted interest, due to their unique features and, particularly, to their usage of unlicensed bands. Several prototypes, devices, and kits based on LoRa are now available, facilitating its deployment and, at the same time, the continuous assessment of their performance.

A meaningful and novel application of these IIoT LPWAN technologies is considered in this paper, involving an additive manufacturing experimental setup realized within the framework of an Italian regional research project, named “ADditive Manufacturing and INdustry 4.0 as Innovation Driver” (ADMIN-4D). The project is a skilful implementation of an IIoT ecosystem, and concerns the design and building of a powder-bed 3D printer of large size (3 m × 3 m × 3 m), and the consequent printing of artifacts for various usages. Artifacts are sensorized at the beginning of the production process, with the aim of monitoring the production phase, as well as the following phase when artifacts are placed in their final deployment sites. Clearly, once the artifacts have been realized, the embedded sensors are not physically accessible anymore. Thus, battery lifetime becomes a key aspect of such an application, together with the capability of the embedded sensors to reliably transmit their data, possibly over long distances.

This research is focused on providing a performance assessment of the system, from the point of view of the reliability and availability of measured data, as well as of the expected useful lifetime. The paper is organized as follows: Section 2 considers relevant previous work and assesses its contribution, highlighting differences with respect to the current state of the art. Section 3 details some essential features of Lora and LoRaWAN. Section 4 describes the test case and considers its features as a meaningful example of an IIoT-based measurement system. Section 5 reports on the experimental assessment and the results obtained. Finally, Section 6 concludes the paper and outlines some future research directions.

## 2. Related Work and Contribution

As a result of the dissemination of IoT technologies, LPWANs and LoRa, in particular, have been extensively considered in recent years. Some interesting contributions have been concerned with the adoption of LoRa for environmental monitoring, often in the context of smart cities. In [10], the authors evaluated the performance of LoRa in a suburban area of Paris. As well as providing an overview of such a network, they implemented a simulation tool to assess criticisms of the protocol, particularly the collision rate behavior. In [11], a water level monitoring system using multiple LoRa devices was described. Notably, the authors introduced a multi-step strategy to guarantee high levels of accuracy and efficiency in data transmission. Both papers [12,13] provided experimental evaluations of the LoRaWAN performance in a specific scenario (a wide and densely populated campus). The results obtained are interesting; however, the analyses carried out by the study authors differ substantially from the assessment presented in this paper in terms of environmental conditions, data collection and testing techniques. In dense networks, due to possible packet collisions, the ALOHA-like protocol adopted by LoRa to access the transmission medium may result inadequate, with consequent negative impact on data reception by the LoRa gateways. In [14,15], this issue is effectively addressed and different solutions are proposed to reduce the number of collisions.

In the industrial context, as is well-known, wireless communication may be further impaired by high levels of noise, as well as by the presence of multiple (possibly moving) obstacles. Such issues are specifically tackled in [16,17,18], which investigated the use of LoRa for IIoT and, in particular, performed preliminary evaluations of the transmission range in this challenging field of applications. In this context, another critical issue is that of power consumption, since LoRa interfaces are often mounted on simple battery-fed sensors. To address this issue, in [19], various battery technologies were considered. The paper highlighted that lithium-based batteries, due to their high energy density, are becoming a valuable choice that can ensure long battery lifetimes. This is confirmed by the assessment carried out in [20], which provides a detailed analysis of the structure of such batteries. In [21], the energy consumption of some LPWAN technologies was considered. The authors provided measurements of the current consumption of devices compliant with some popular networks, such as LoRaWAN, DASH7, Sigfox, and NB-IoT, and estimated their respective battery lifetimes. The results of the analysis were compared with those obtained from a LoRa network used to connect environmental sensors in a greenhouse.

The configuration parameters of LPWAN nodes may impact on power consumption and energy efficiency. In [22], the authors addressed this issue by deploying LoRa nodes in an outdoor area and analyzing the impact of different configurations of both media access control (MAC) and physical (PHY) layers on battery lifetime. In particular, the lifetime of different battery chemicals, namely Li-Ion and Ni-Cd, was considered. In this respect, the battery typology is of considerable importance, since it has a direct impact on performance. A study of battery life duration was also reported in [23,24]. The authors developed a model to precisely describe the energy consumption of a general wireless network, and then compared the behavior of some widespread IoT protocols in terms of battery dissipation. Moreover, in [25], a valuable probabilistic battery consumption model for LoRa devices was developed. Notably, the energy drain in terms of the power required to transmit or receive a LoRa packet was modeled, starting from a precise analysis of the sensor battery lifetime, based on different operational modes (active or sleep). The authors developed a model fitting with non-constant battery consumption, typical of LoRa devices (and, more generally, of LPWANs) and validated it via an experimental strategy.

From the above analysis, and to the best of the authors’ knowledge, there are no studies that have investigated the behavior of smart and IoT sensors embedded within 3D artifacts. This paper moves away from the considerations emphasized above and, instead, addresses the ADMIN 4D project, which is focused on the implementation of sensorized artifacts, as well as on the consequent acquisition of sensor data. In this respect, from a practical point of view, sensors and artifacts become part of a smart industrial measurement system within the IIoT ecosystem represented by the whole ADMIN-4D project. Undoubtedly, the materials with which the artifacts are built might compromise the functionality of the sensors, or impact on their transmission range. Moreover, the artifacts may be deployed in places with severe variations in temperature, humidity, and pressure that could affect smart sensor performance and, in particular, the lifetime of the batteries.

The primary contribution of this paper is the investigation of the suitability of the proposed design for the embedded sensors, specifically focusing on their ability to reliably transmit sensor data. From this perspective, transmission ranges, battery lifetime and energy efficiency are carefully addressed. Notably, to validate battery discharge curves, the testing strategy presented spans over long time intervals, and uses sensor transmission periods specifically selected for the application.

## 3. LoRa and LoRaWAN

LoRa was developed by Semtech, and its physical layer serves as the foundation for LoRaWAN. It operates in industrial, scientific, and medical (ISM) bands that, in Europe, correspond to 867 MHz– 870 MHz. LoRa uses a chirp spread spectrum (CSS) modulation to encode data, and each transmission can be characterized by tuning two main parameters, namely, a spreading factor (SF) and bandwidth (B). The SF varies from 7 to 12, while B can be set to either 125 kHz or 500 kHz. Both such parameters allow definition of the duration of each symbol as $\frac{1}{B}*2^{SF}$. High values of SF lead to improved noise immunity. This occurs, however, at the expense of the transmission rate, which is reduced due to the increased symbol duration.

**Figure 1:**
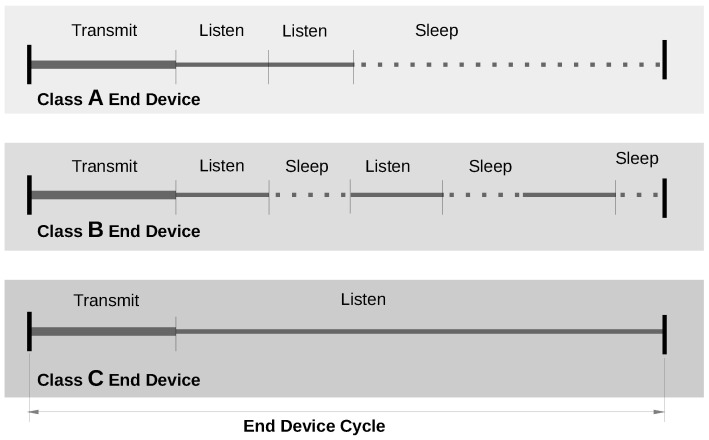
LoRa end devices classification scheme.

A LoRaWAN network relies on a star topology and comprises three types of devices: end devices (EDs) that serve as LoRa sensors, gateways (GWs), and a network server (NS). EDs are the data gatherers that send their information to one or more gateways in their range, using an ALOHA protocol to access the wireless medium. A GW receives a LoRa packet and forwards it to the network server that may either accept or discard it. In this way, most of the computational burden is handled by the NS, thus simplifying the complexity of the other devices (specifically EDs), with consequent benefits, particularly in terms of power consumption. In this regard, as described in [26], the job cycle of EDs is split into three parts, namely transmit, listen and sleep, as shown in Figure 1, which reflect the respective operational modes of the node. The beginning of a cycle is determined by beacon messages sent by the GWs, which, in turn, are synchronized by the NS. The end devices are grouped into three different classes: All (A), Beacon (B), Continuous (C). The three classes have a common transmit phase in which nodes can randomly access the network to transmit their data, whereas they differentiate on the subsequent phases. After transmission, Class A EDs open one or two consecutive temporal listen windows, in which they can receive messages coming from the NS. Then, class A EDs enter the sleep mode. Class B EDs extend such behavior, potentially opening more listening windows at scheduled times. Finally, Class C EDs, after transmission, enter the listening mode which is maintained until the end of the cycle. In practice, the sleep mode is never entered by such EDs. Clearly, moving from Class A to Class C, the power consumption of EDs increases.

In a typical LoRa network, several EDs may contemporaneously forward data through the gateway to the network server. For this reason, one of the main issues related to LoRa is the high collision probability of packets coming from different EDs. In this context, the SF plays a key role, as each SF level defines a quasi-orthogonal transmission channel with respect to the others. In this way, six independent channels can be defined, so that, by tuning the spreading factor of six adjacent EDs, it is possible to avoid collisions without any particular strategy. If the usage of the same SF for two or more EDs is unavoidable, the adoption of effective strategies to limit collisions is needed. One of the most popular solutions is the application of a “listen before talk” (LBT) strategy to prevent the transmission of a message if the network is not idle, even if, as specified in [27], this technique increases power consumption.

Finally, it is important to note that there are two ways for an ED to connect to a network, namely, over-the-air activation (OTAA) and activation by personalization (ABP). OTAA relies on a handshake procedure between the ED and the NS, started by the ED which requires to join the network. Conversely, ABP makes use of a pre-registration of the ED carried out during network setup. Then, the ED can be included at any time upon a request issued either by itself or by the NS. In this latter case, the NS can change the period with which data is transmitted by the ED. This is an important feature in the context of the ADMIN 4D project, since it allows remote adaptation of the transmission periods of the sensors, depending on the operational phase in which the artifacts are involved, i.e., either production or final deployment.

## 4. Additive Manufacturing Application

**Figure 2:**
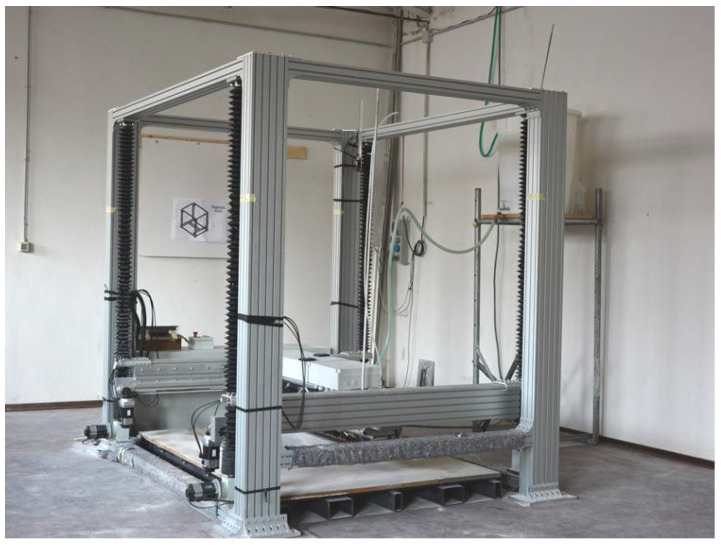
The 3D-Printer employed in the ADMIN 4D project.

The ADMIN 4D project is an IIoT ecosystem based on a large powder-bed 3D printer, like that shown in Figure 2 for the additive manufacture of concrete-like artifacts. The additive manufacturing technology is based on the composition of layers of dusts and natural bindings that are solidified due to a chemical reaction. Several types of materials can be employed for artifact construction, because the bindings are conceived to react with a wide range of diverse components. As an example, marble powder and river/sea sand have been effectively used in preliminary tests of the printer. This achievement is particularly beneficial and interesting for the whole project, since it reflects the very limited environmental impact of the production phase. Recycled materials, such as the wastes of marble processing, can be used after they are shredded and reduced to powder, giving new life to materials that, otherwise, would have been disposed of.

Every printed piece is first designed using 3D modeling software and subsequently transferred to a 3D-printer. The entire operation of the printer is handled by an Omron NX102-9020 PLC. In particular, it is of primary importance that all the phases in the lifespan of an artifact can be monitored in a continuous and reliable way, from the production phase to final positioning in the deployment site. To this extent, as mentioned, during the first printing steps, some smart sensors are totally embedded within the artifacts. The sensors are equipped with LoRa interfaces and represent end devices of a LoRaWAN network. To ensure sensor integrity, the sensors are positioned within adequate IP67 certified enclosures during the production phase. Depending on the sensor type, the antenna may be placed either inside or outside the enclosure but, in any case, are embedded in the artifact. With this strategy, the sensors are not externally visible or accessible. Clearly, to improve the transmission range, alternative solutions could be envisaged that allow the antenna to be positioned outside the artifact. However, this was not among the goals of the project that, conversely, relies on the complete autonomy of the artifacts. As a consequence, such an option has not been investigated. Moreover, and more importantly, the experimental results shown in the next section clearly demonstrate that the measured transmission range of the embedded sensors adequately fits the project requirements.

The specific nature of the application, with sensors becoming totally unreachable after the production phase, raises some significant issues: nodes maintenance is impossible, batteries can neither be replaced nor recharged, and wireless connectivity is the only viable solution for data transmission and further configurations.

**Figure 3:**
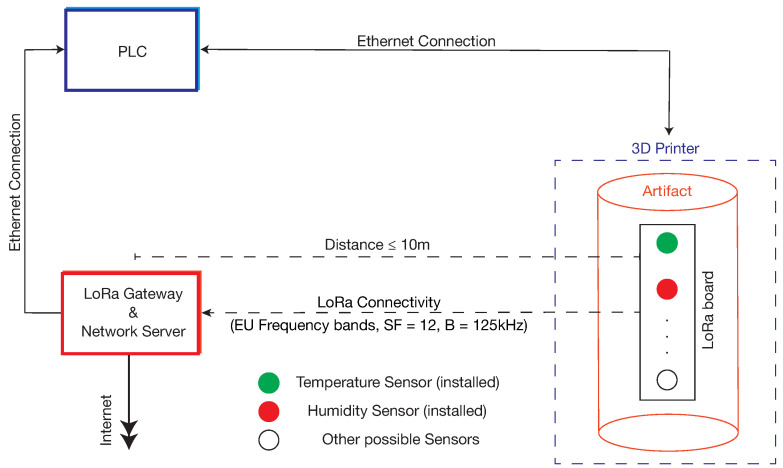
Sensor data transmission in the artifact production phase.

In the artifact production phase, the embedded sensors transmit measurement data, typically temperature and humidity, to the gateway and network server. All nodes of the LoRaWAN network are placed close to each other, typically in the range of a few meters. Figure 3 shows the configuration of the ADMIN 4D components involved in the production phase. As can be seen, the PLC receives data from the gateway, connected to it via an ethernet interface. In addition, the gateway is connected to the Internet for the transmission of sensor data to a remote cloud.

The whole production phase duration depends on the artifact size. However, considering the high number of layers, and that printing an area of 1 m^2^ takes about 45 s, the process of printing an artifact typically takes 4–5 h. Consequently, the period for sending the sensor data can be safely set to some minutes, so that anomalous variations of production variables can be detected in a timely way and adequate corrective actions effectively undertaken by the PLC. Two crucial variables are monitored during the production phase, namely temperature and humidity, since they need to be kept within specified ranges, related to the features of both the binding and the material employed. However, other sensors could be added in the future, depending on production requirements.

**Figure 4:**
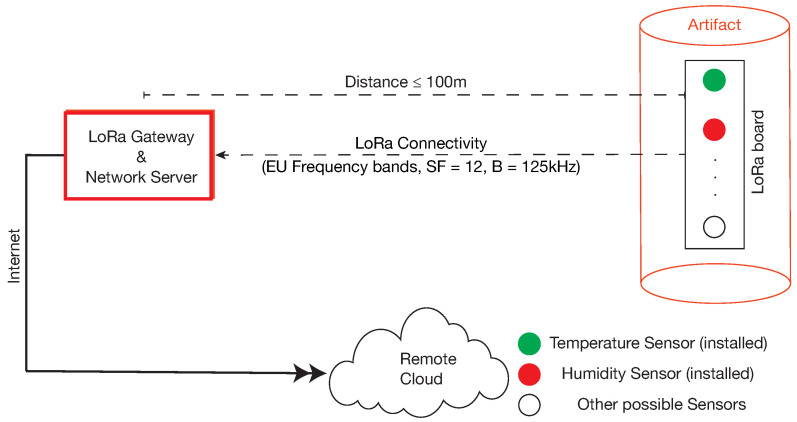
Sensor data transmission in the artifact final deployment phase.

After the production phase, artifacts are placed in their final deployment sites, from which they continue to transmit sensor data. The new system architecture of data transmission is depicted in Figure 4. The EDs, gateway and network server are placed at a distance of some tens of meters. Sensor data is transmitted to the remote cloud by the gateway, which is connected to the Internet. This data is used for artifact monitoring as well as for offline analyses. Monitoring is necessary to detect possible artifact anomalies, especially during the first period after the final deployment. Additional sensors may be used for this activity; for example, strain gauges may be very helpful for the detection of unexpected artifact deformations. Clearly, such sensors have to be inserted during the production phase, but may be (remotely) activated later on. Offline analyses allow implementation of a kind of “long–term feedback”, since the outcomes will be used to tune the parameters of future artifact production. For both these activities, the typical requirements for the communication system are extended transmission ranges, high reliability, low power consumption, and sending periods that range from tens of minutes to hours. To obtain meaningful results, the sensors have to transmit their data for a long time after artifact deployment. Thus, a further important requirement relates to the lifetime of the batteries, which, for the ADMIN 4D project, was set to a minimum of one year.

Table 1 summarizes sampling periods and typical distance ranges in the two operational phases.

Finally, it is worth noting that sensorized artifacts are envisaged to be used in several fields of application, such as smart cities [28], street and civil furnishing and the monitoring of cultural heritage. For example, very small sensorized artifacts may be inserted in the wall of ancient buildings to monitor their hygrometric status via humidity measurements. In the context of smart cities, one of the partners of the ADMIN 4D project, namely Desamanera [29], is involved in the design and implementation of smart park benches, equipped with sensors of various types that will allow monitoring, not only of environmental variables (e.g., temperature and humidity), but also of the presence of people, with the aim of assessing, for instance, the crowding of some locations.

## 5. Experimental Setup and Results

**Figure 5:**
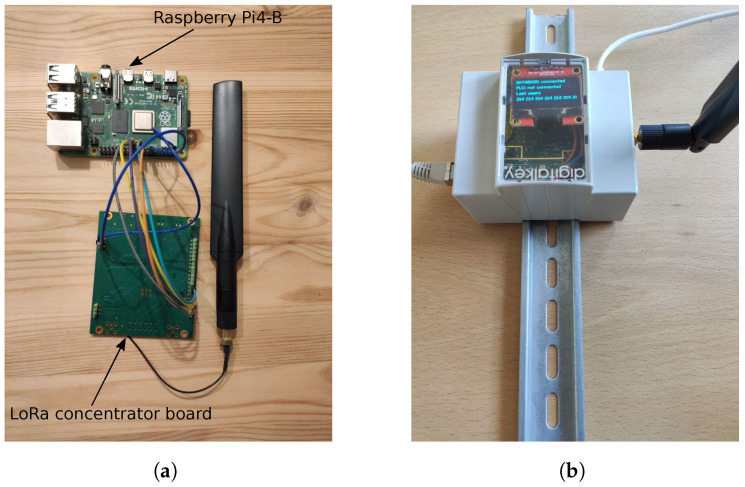
LoRa gateway and network server. (**a**) Spare GW/NS components. (**b**) Packed GW/NS components.

The LoRaWAN network for the described application comprises the gateway, the network server, and a set of EDs embedded into the artifacts. Both the GW and the NS have been implemented in a Raspberry Pi 4 model B leveraging on an open-source LoRaWAN network server stack, namely, ChirpStack. The system design makes use of an iC880A LoRa concentrator board, that forwards the LoRa packets coming from the EDs to the Raspberry through the serial peripheral interface (SPI). Figure 5 shows the spare components used for the gateway and network server. The Raspberry platform was selected because of its flexibility, allowing the hosting of all of the ChirpStack utilities, as well implementation of ad hoc algorithms for the effective transfer of the LoRa packets.

As far as the EDs are concerned, to obtain a meaningful assessment of battery lifetime and transmission range, two distinct devices were considered:

- Microchip RN2483 LoRa Mote;
- Tinovi PM-IO-5-SM LoRaWAN IO Module;

The first [30] is a fairly classic “mote”, i.e., a standalone battery-powered node, that features all the needed circuitry, interfaces and connections to realize a LoRaWAN Class A ED in the 868 MHz band, based on the Microchip RN2483 LoRa modem certified by the LoRa alliance. The mote is equipped with two sensors: an Everlight Electronics ALS-PT19-315C/L177/TR8 ambient light sensor, with a wavelength range of 390 nm to 700 nm, and a low-power linear active thermistor, namely Microchip MCP9700, for temperature measurements between −40 ${}^{\circ }C$ and 150 ${}^{\circ }C$ with an accuracy of 1 ${}^{\circ }C$. This ED can be programmed employing an external host computer through a convenient USB connection.

On the other hand, the Tinovi is a different type of device. It is designed and implemented in a proprietary design, whose purpose is to provide an effective platform for soil moisture measurement, through the PM-WCS-3-I2C sensor that can measure extrinsic properties of the material in which it is embedded. The realization of this smart sensor is accomplished by adding a communication stage that again implements a LoRaWAN Class A end-device. The producer provides a reference datasheet [31], reporting the technical information for the soil moisture sensing performance. Unfortunately, the information about the communication interface is rather poor. Nevertheless, it is also possible to analyze the corresponding source code repository, publicly available on a popular website, and infer that the Tinovi ED exploits a microchip platform, this time based on the SAMR34 IC, which is not a simple LoRa modem, but also includes an integrated 32-bit Arm Cortex-M0+ based MCU, with a sub-GHz radio and proven LoRaWAN software stack.

The main characteristics of the aforementioned devices are summarized in Table 2. These two EDs have been chosen on a preliminary basis as potential candidates for the ADMIN 4D project, due to their flexibility, usability and performance, notwithstanding that they are characterized by a significant difference from a communication perspective, that is, the potential availability of an external antenna. Indeed, the Tinovi module features a dust and waterproof IP68 design, that does not allow the use of external antennas, forcing the use of the built-in antenna. Conversely, the Microchip mote is a node designed for showcasing increased degrees of freedom for testing and development in a broader set of applications. Although unable to provide any protection from water/dust, it supplies improved interfacing, and allows the connection of different kinds of external antennas. Clearly, different devices could have been adopted for the experimental strategy. For example, several types of LoRa EDs, equipped with sensor types, antennas and batteries (potentially) suitable for the ADMIN 4D project, are available off-the-shelf. However, the activity carried out was not meant as a comparison among device types but, rather, as a feasibility study of the proposed IoT based measurement system. For this reason, the assessment was performed on a limited number of devices. Based on the findings of this study, future development will include more comprehensive analyses involving several different products.

Using the hardware described, an extensive experimental campaign was carried out aimed at assessing the suitability of the chosen EDs for the project, particularly in terms of (i) guaranteed coverage range, and (ii) expected battery lifetime. A description of the tests undertaken and discussion of the relevant results are provided in the following paragraphs.

### 5.1. Coverage Range Tests

The goal of this set of tests was to assess the coverage range of the chosen sensors considering both the phases that characterize the application, production and final deployment, which highlight different issues from a communication point of view.

In the first test, an external Taoglas TI.08.C.0112 antenna [32] was employed for the Microchip sensor, whereas the built-in antenna was used for the Tinovi. The goal was to investigate the behavior of the transmission even in highly critical scenarios, typical of the production phase, which can be represented by an indoor mixed industrial/office settlement where several electronic devices (e.g., smartphones, computers, 3D printers, etc.) are present, together with multiple walls, metallic structures and objects that may occlude the line of sight. A very good candidate for these tests was represented by one of the research building laboratories, located at the Department of Information Engineering, University of Padova (Italy), that hosts several laboratories, offices, and industrial machines over several floors, and hence meets the previous conditions. During the tests, the operational conditions of the production phase were emulated as much as possible. For this reason, the EDs were inserted in a box containing the same material used for the artifacts (river sands in this case), even if they were not progressively embedded, as occurs during the actual production phase. The LoRa GW and NS were placed inside the laboratory so as to have direct control of them, while the EDs were moved to several different locations within the Department at different distances and floors.

The experiments were performed with both the EDs, and adopting the same spreading factor, SF=12. For a transmission test to be considered as passed, the LoRa gateway had to receive at least three-quarters of the transmitted data without errors, a proportion that has been considered to be able to ensure sufficient measurement continuity in the context of the ADMIN 4D project. The obtained results are reported in Table 3, where “Yes” and “No” account for test passed or not, respectively.

The results showed that, on the one hand, LoRaWAN transmissions were quite reliable even in harsh environments, confirming the results obtained in other independent assessments [33], where robustness of the network over the 868 MHz band for indoor operations was highlighted. On the other hand, the experiments showed, as expected, the more limited coverage range of the Tinovi sensor. Indeed, this sensor was unable to reliably receive packets beyond a range of 10–15 m, whereas the Microchip mote performed reliably within a 70 m range in the indoor experiments.

Nevertheless, it must be highlighted that both sensors, by comparison with Table 1, were able to meet the requirements for the production phase scenario, where the maximum expected coverage range is less than 10 m.

Further tests were conducted with the aim of evaluating the coverage range of the sensors after they had been embedded into the produced artifacts, reflecting the final deployment phase. In these cases, the sensor module was directly surrounded by the building material and placed at higher distances than in the previous case, typically in outdoor locations.

**Figure 6:**
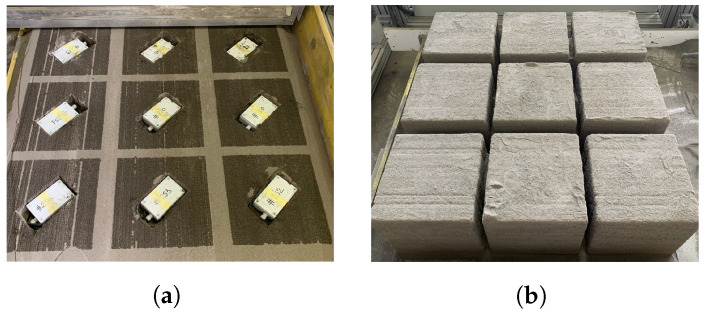
Two steps of the production phase with the embedding of Tinovi sensors. (**a**) Sensor embedding during production phase. (**b**) Final results.

For this experimental setup, the tests concerned only the Tinovi smart sensor, since the Microchip LoRa mote had already been assessed in a recent study [34] under analogous conditions, and was demonstrated to be able to reach distances of more than 100 m when embedded into a sample artifact. Therefore, to perform the tests, the Tinovi sensors were embedded in a composite of river sand and binders like the final substrate used for the printing process. Figure 6 illustrates two steps of this process relevant to the printing of nine cubes of 40 cm side.

**Figure 7:**
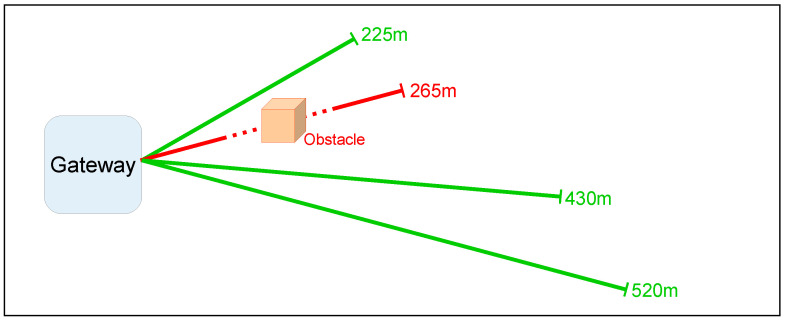
Tinovi sensor range tests 1: the industrial environment.

Once the artifact was devised, the LoRa gateway was positioned inside the main ADMIN–4D office and the ED was placed in four different outdoor locations to evaluate its transmission behavior. As in the previous tests, the experiments were performed with the same spreading factor, SF=12; transmission tests were considered as passed when the GW received at least three-quarters of the transmitted data without errors. The results are presented in Figure 7, where the green lines stand for successful trials, while the red line indicates a failure in the transmissions.

The analysis of Figure 7 highlighted that the Tinovi ED was able to quite efficiently covera range of 500 m in the absence of obstacles. The range was more limited in the case of strong obstructions in the line of sight path. Nevertheless, the final result was that the Tinovi ED was shown to be able to meet the coverage range requirements of the application.

### 5.2. Power Consumption and Battery Lifetime Estimation Tests

This subsection is concerned with the experimental activities devised to analyze the power consumption and battery lifetime of the selected LoRa EDs.

Neither the Microchip nor the Tinovi documentation provided the necessary specifications in terms of power consumption or absorbed current in the different working modes, i.e., either in sleep or idle state, and during transmission/reception. Nonetheless, as for both the EDs an indication about the transceiver model was available, it was possible to carry out a preliminary comparison, as shown in Table 4.

A first set of measurements was then performed to evaluate the lifetime of sensors when they are equipped with low-cost, off-the-shelf, battery types. In this preliminary analysis, only the Microchip LoRa Mote was tested. This represents a type of worst case, since this mote cannot be fed with recent high energy density and capacity batteries, allowing the use only of classic AAA alkaline or rechargeable batteries. The tests were carried out using alkaline batteries from two different manufacturers [35,36]. The parameter “shelf life”, which represents an indication of the time the batteries will hold their charge without being used when stored under normal environmental conditions, was different for the two brands, being equal to five and ten years, respectively. This, however, represented a very low self discharge rate, and, considering the expected run time for the mote in this application, it may be argued that the final lifetime is almost exclusively dependent on the energy efficiency of the mote.

In the measurement campaign, the battery lifetime was estimated as the time elapsed between the instant the module was powered on and the timestamp of the last received LoRa packet. The Microchip LoRa mote does not provide any means to directly measure the charge status of the batteries, and, consequently, it was not possible to precisely track the discharge curve. Clearly, a significant parameter impacting on the lifetime is the sampling period required by the embedded sensor. Therefore, tests were performed in agreement with the values indicated in Table 1. The results are provided in Table 5.

As expected, the impact of the sending period on the battery lifetime was considerable. Moreover, batteries with longer shelf-life performed significantly better than their counterparts. Nevertheless, the increase in lifetime was not linearly dependent on increase in the sending period. This might be related to a lower energy efficiency of the mote when no transmission occurs, and specifically to a non-optimal management of the energy saving states available for the LoRa chip (i.e., switching between idle and sleep states) and the MCU.

Before presenting the next set of measurements, it is worth providing some observations regarding the available LoRa sensor nodes. From the assessments carried out to this point, the Microchip LoRa motes were not completely suitable for deployment in the context of the ADMIN 4D project. As already pointed out, such motes can not host battery types other than basic AAA batteries, preventing the assessment of more recent battery technologies. Moreover, these modules do not offer the possibility of remotely changing their transmission parameters, limiting the potential for fine tuning of the data rate and sending period. Finally, the Microchip motes require an enclosure size that, in some cases, may be larger than what is allowed by the application. Considering the worst case situation, i.e., the smallest possible artifact to be realized by the additive manufacturing application, the Microchip mote resulted in a sealed device which was unfortunately larger than the imposed bounds, since it needed a suitable external enclosure for both the mote and its external antenna [34].

Therefore, in the next assessment, only the Tinovi LoRa ED was addressed since it demonstrated its compliance with the ADMIN 4D requirements. It can also be fed by more recent battery types characterized by more advanced chemistry than classic alkaline batteries. Moreover, it allows for a remote set-up, i.e., connection and transmission parameters can be dynamically changed even when the end devices are in movement or are physically unreachable. Additionally, the Tinovi sensor allows transmission of information about battery state of charge, ranging from 2.8 V to the maximum voltage reached during the charging (4.2 V) with a resolution of 1%. This feature is of particular importance for characterizing the behavior of batteries, as well as accurately comparing different battery typologies in terms of lifetime.

The following set of measurements are, therefore, devoted to a performance analysis of more recent high capacity batteries in this additive manufacturing scenario. Two different battery types were adopted: the first was an MKC 18,650 lithium-ion battery, while the second was a SAFT LS 17,500 lithium thionyl chloride battery. Their specifications are summarized in Table 6. Both these technologies tend to became unstable (i.e., risk of explosion or fire) when used outside the operating temperature range, and this has to be carefully taken into account because the temperature reached inside the artifacts during the production phase may be quite high due to binder reaction processes. Nevertheless, a preliminary extensive analysis highlighted that, due to the IP67 enclosure, the battery operating temperatures always remained within the maximum allowed values.

The two different types of chemistry that were selected for this analysis are notably different, each possessing strengths and weaknesses. Lithium-ion (Li-ion) batteries have become the standard for consumer electronics. They are characterized by low cost, high availability and, most importantly, they can be recharged. However, in general, this type of battery suffers from a relatively high self-discharge rate and is strongly dependent on the discharge curve with temperature [39].

These limitations do not apply to lithium thionyl chloride (LTC) batteries. These are primary cells, therefore not rechargeable, capable of delivering low currents, but characterized by both a flat discharge curve, in the range of the nominal voltage, and low dependence on the operating temperature [40]. These characteristics make them particularly suitable for the ADMIN 4D project and, more generally, for supplying battery powered IoT measurement devices deployed in adverse environments.

**Figure 8:**
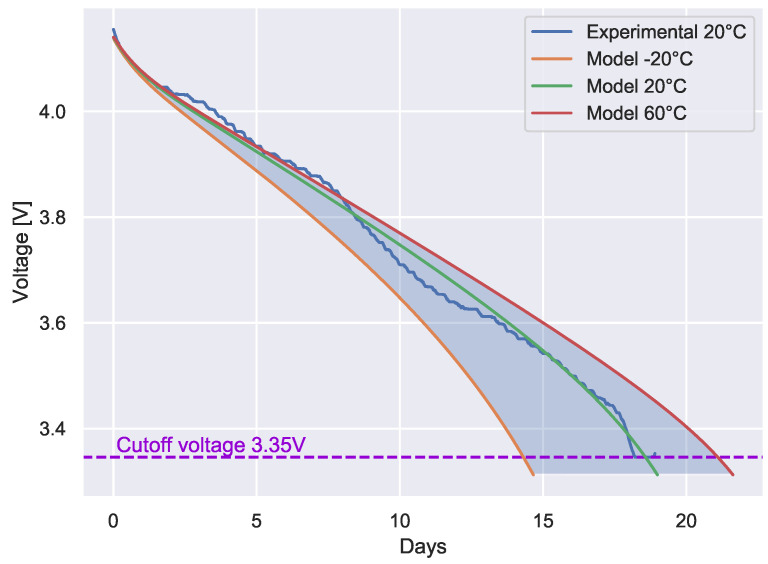
Tinovi PM−IO−5−SM with Li−ion batteries: discharge curves obtained from both experimental measurements and theoretical model, with a transmission period of 5 min.

In the following measurement approach, a transmission period of 5 min was adopted, representing a value typically adopted in the production phase of an artifact In the first set of measurements, Li-ion batteries were considered. A Tinovi ED was periodically sampled to obtain information about battery voltage, to derive the discharge curve. This is represented by the continuous blue line in Figure 8.

From the figure, it can be observed that the employed Li-ion batteries ensured continuous and reliable operations for 18 days, after which the battery voltage dropped below the cutoff voltage. A further observation was that the obtained duration and discharge curve partially refuted the preliminary linear estimation model for Li-ion batteries proposed in [41]. Thus, in order to achieve a better estimate of the battery lifetime for different temperature ranges, the popular model described in [42,43] was adopted (the same model is implemented in the Mathworks Simscape suite).

This model provides an estimate of the Li-ion battery voltage as

(1) $$V_{batt}\left(T\right)=f_{1}\left(i,T,T_{a}\right)-R\left(T\right)\;\cdot \;i$$

that is, the voltage is a (decreasing) function of the discharge current *i*, as well as of the ambient and internal battery temperatures *T* and $T_{a}$, respectively. The second term of Equation (1), R(T), represents a discharge resistance dependent on the temperature equivalent to the thermistor model.

The function $f_{1}$ contained in Equation (1) contains several terms that can be derived from the analysis of the battery specifications. The model equation is

(2) $$f_{1}\left(it,i^{*},i,T,T_{a}\right)=E_{0}\left(T\right)-K\left(T\right)\;\cdot \;\frac{Q(T_{A})}{Q(T_{A})-it}\;\cdot \;\left(i^{*}+it\right)+A\;\cdot \;exp\left(-B\;\cdot \;it\right)-C\;\cdot \;it$$

Some main terms can be identified in Equation (2), corresponding to the different typical discharge phases of a battery. In particular, a temperature-dependent initial voltage $E_{0}\left(T\right)$ (fully charged) is followed by an exponential discharge phase, and by a nominal discharge phase. The description of the terms in Equation (2) is reported below

- $E_{0}$ is the voltage at the end of the exponential region, called the constant voltage (V)
- *Q* is the maximum battery capacity (Ah)
- *K* is the polarization constant (V/Ah), often indicated as polarization resistance (Ω)
- *i* is the battery current (A)
- $i^{*}$ is a low-pass filtered version of the battery current, characteristic of this type of batteries, that often can be considered equal to *i* (A)
- it=∫idt is the actual battery charge (Ah)
- *A* is the exponential voltage (V)
- *B* is the exponential capacity (A/h)
- *C* is the nominal discharge curve slope (V/Ah)
- *T* is the cell or internal temperature (K)
- $T_{a}$ is the ambient temperature (K)

A detailed analysis of the models, as well as an in-depth explanation of the different terms, and the way they can be obtained from the battery data sheet, can be found in [42].

**Figure 9:**
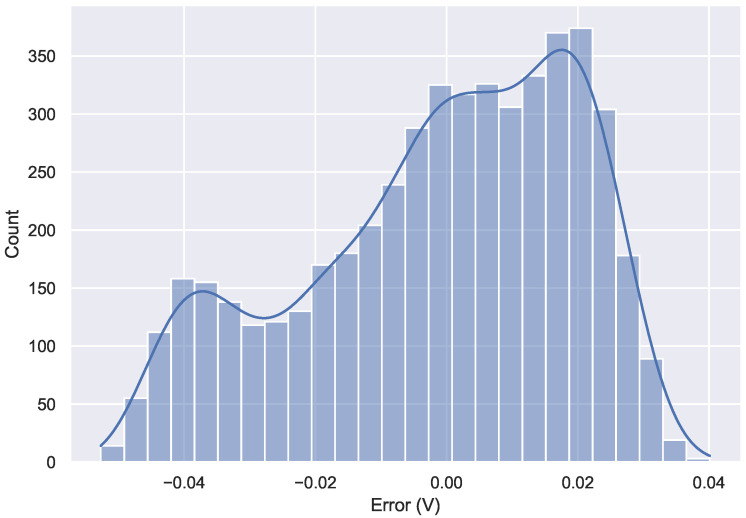
Density function of the model calibration error.

To obtain realistic results, the model parameters were tuned using the results of the experimental measurements carried out at 20 ${}^{\circ }C$. Then the model was calibrated to match the boundaries of the discharge curve and to minimize the error in the central part. The probability density function of the calibration error is shown in Figure 9. As can be seen, the calibration error was limited to the range −0.05 V–0.04 V, making it suitable for the intended use.

The calibrated model was then used to simulate the discharge curves for different temperature ranges. The results are reported in Figure 8. As can be observed, the battery lifetime was strongly dependent on the operating temperature.

**Figure 10:**
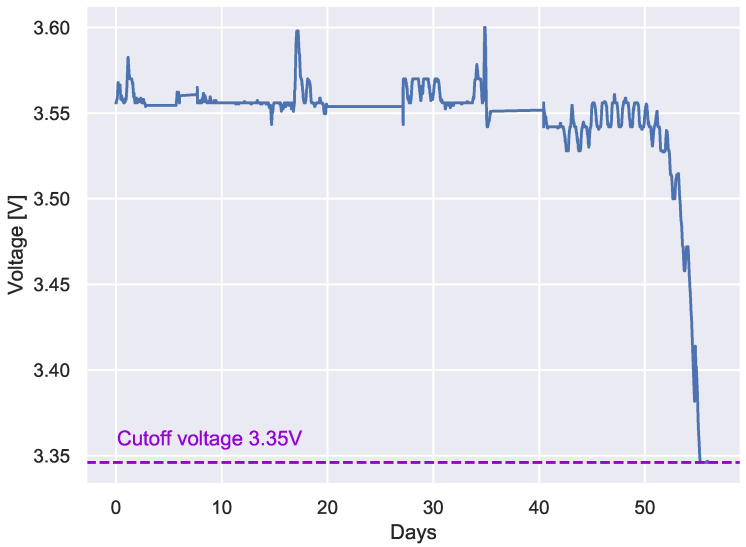
Tinovi PM-IO-5-SM battery lifetime estimation. Experimental discharge curve for a transmission period of 5 min and LTC battery.

A second set of measurements was carried out considering the SAFT LS 17,500 LTC battery, using the same configuration and sending period for the previous experiment. The whole experimental campaign lasted about two months, and the results obtained are shown in Figure 10.

The plot evidences the almost flat discharge curve characterizing the lithium thionyl chloride batteries. The batteries maintained a rather stable voltage of 3.56 V for nearly 40 days, a value very close to the 3.6 V nominal voltage. After this period, the voltage started to oscillate around 3.55 V. Then, after 50 days, the voltage commenced a quite abrupt decay and, in three to four days, reached the cut-off voltage where the sensor powered off.

As can be seen by comparing Figure 8 and Figure 10, the use of a LTC battery significantly increased the lifetime of the sensor.

It should be noted that the experiments with the two battery types were carried out with a sampling period of 5 min. This period is used in the production phase that typically lasts only a few hours, whereas in the final deployment phase, the sampling periods will be significantly longer. Thus, although a simple linear regression to predict the battery lifetime cannot be applied, it may be argued that, using a sampling period of the order of some hours (typical for the final deployment phase), the Tinovi sensor would be expected to last for a considerable amount of time (likely more than a year), which is a satisfactory lifetime for the ADMIN 4D project.

## 6. Conclusions and Future Directions

In this paper a novel IoT measurement scenario was considered, where a LoRaWAN network was exploited to gather measurements from embedded sensors in the context of additive manufacturing. Both extensive analysis and an experimental campaign were described, with the aim of characterizing the coverage range and the battery lifetime performance of the chosen smart sensors. With respect to the coverage range, both the analyzed sensor nodes satisfied the application requirements. The outcomes revealed a satisfactory transmission distance for the tested devices, both in indoor harsh conditions (resembling those found in the artifact production phase) and in outdoor scarcely obstructed contexts, like those typical of the final deployment phase. Focusing on the battery lifetime, classic alkaline batteries on the microchip mote provided acceptable results, but were not completely satisfactory and were strongly dependent on the producer and the battery shelf-life. For the application considered, the use of Li ion batteries is discouraged, since they had quite a short lifetime. The most significant results were obtained for the lithium thionyl chloride batteries, when tested on the Tinovi sensor. These batteries showed a lifetime significantly longer than that for the Li ion or alkaline batteries. Moreover, they evidenced a quite flat discharge curve, making them particularly suitable as a power supply solution for IoT LoRa sensors.

The concepts addressed by this paper are novel with respect to the current state of the art. The embedding of sensors within 3D-printed artifacts, and the relevant measurement system to gather sensor data developed in the IoT context, were not, to the best of the authors’ knowledge, formerly considered by the scientific community. The work described here may pave the way for several future activities. First and foremost, it is necessary to extend the analysis to other IoT LoRa devices, especially focusing on other LoRa chips, antenna configurations and considering other types of batteries and sampling periods. A second, but equally important area of development concerns the parameter adaptation capabilities of LoRaWAN, as an effective means of enhancing network performance in a harsh additive manufacturing scenario, for instance, through suitable adaptive data rate (ADR) strategies. This research direction appears promising in this context, since it may further improve network reliability and sensor lifetime, and widen the spectrum of applications. The end of an artifact’s life cycle constitutes one of the most salient issues in this project. In the future, wireless charging strategies will be investigated to determine if such technologies may constitute a suitable solution to the problem, or if the adopted materials affect the recharging process. Another challenging research direction is the definition of effective models for the high accuracy estimation of residual battery lifetime. This would represent a significant step forward in an IoT measurement scenario; an accurate charge status estimation might be used as feedback information for smart sensor parameter adaptation (to tune energy saving strategies, for example), to improve both the continuity and reliability of measurement activities.

## Figures and Tables

**Table 1:** Transmission requirements for the two operational phases.

Phase	Sampling Period	Distance
Production	≤5 min	≤10 m
Final Deployment	≥60 min	≤100 m

**Table 2:** Features of the two EDs used in this work.

	Microchip RN2483 LoRa Mote	Tinovi PM-IO-5-SM LoRaWAN IO Module
LoRa radio	RN2483	SAMR34
MCU	PIC18LF45K50 8 bit - 32 KB Flash	32-bit Arm Cortex-M0+
Programming Interface	USB Micro-B Connector/Ext. PC	USB Micro-B Connector + Android APP
Antenna	External—SMA connector	Built-in
Enclosure	NO	IP 67
Sensors	- MCP9700 – Linear Active Thermistor	- PM-WCS-3-I2C soil moisture sensor
	- Everlight (ALS-PT19-315C) Ambient Light Sensor	

**Table 3:** End Devices transmission comparison.

Distance (m)	Microchip RN2483 LoRa Mote	Tinovi PM-IO-5-SM LoRaWAN IO Module
2	Yes	Yes
10	Yes	Yes
45	Yes	No
70	Yes	No

**Table 4:** Current absorbed by the transceivers of the two EDs employed in this study at 3.3 V.

Working Mode	RN2483 LoRa Chip	SAMR34 LoRa Chip
Sleep	1.3 μA	0.79 μA
Active	2.8 mA	1.4 mA
TX/RX	38.9/14.2 mA	32.5/14.8 mA

**Table 5:** Microchip LoRa Mote battery lifetime results.

Shelf Life (years)	TX Period (min)	Lifetime (days)
5	5	27
	60	32
10	5	55
	60	77

**Table 6:** Summary of the main battery characteristics.

a. MKC 18,650 battery specification [37]	
**Description**	**Specification**
Rechargeable	Yes
Nominal Voltage	3.70 V
Standard Capacity	2000 mAh
Measured Cut-off Voltage	3.1 V
Operating Temperature	−20 °C to 60 °C
b. SAFT LS 17,500 battery specification [38]	
**Description**	**Specification**
Rechargeable	No
Nominal Voltage	3.60 V
Standard Capacity	3600 mAh
Measured Cut-off Voltage	3.3 V
Operating Temperature	−60 °C to 85 °C
